# Clinical predictive modelling of post-surgical recovery in individuals with cervical radiculopathy: a machine learning approach

**DOI:** 10.1038/s41598-020-73740-7

**Published:** 2020-10-08

**Authors:** Bernard X. W. Liew, Anneli Peolsson, David Rugamer, Johanna Wibault, Hakan Löfgren, Asa Dedering, Peter Zsigmond, Deborah Falla

**Affiliations:** 1 School of Sport, Rehabilitation and Exercise Sciences, University of Essex, Colchester, Essex, UK; 2 Department of Health, Medicine and Caring Sciences, Division of Prevention, Rehabilitation and Community Medicine, Unit of Physiotherapy, Linköping University, Linköping, Sweden; 3 Department of Statistics, Ludwig-Maximilians-Universität München, Munich, Germany; 4 Chair of Statistics, School of Business and Economics, Humboldt University of Berlin, Berlin, Germany; 5 Department of Activity and Health, and Department of Health, Medicine and Caring Sciences, Linköping University, Linköping, Sweden; 6 Neuro-Orthopedic Center, Jönköping, Region Jönköping County, Sweden; 7 Department of Biomedical and Clinical Sciences, Linköping University, Linköping, Sweden; 8 Allied Health Professionals Function, Department of Occupational Therapy and Physiotherapy, Karolinska University Hospital, Stockholm, Sweden; 9 Department of Neurobiology, Care Sciences and Society, Division of Physiotherapy, Karolinska Institutet, Stockholm, Sweden; 10 Department of Neurosurgery, Linköping University Hospital, Linköping, Sweden; 11 Centre of Precision Rehabilitation for Spinal Pain (CPR Spine), School of Sport, Exercise and Rehabilitation Sciences, College of Life and Environmental Sciences, University of Birmingham, Birmingham, UK

**Keywords:** Diseases, Health care, Medical research, Risk factors

## Abstract

Prognostic models play an important role in the clinical management of cervical radiculopathy (CR). No study has compared the performance of modern machine learning techniques, against more traditional stepwise regression techniques, when developing prognostic models in individuals with CR. We analysed a prospective cohort dataset of 201 individuals with CR. Four modelling techniques (stepwise regression, least absolute shrinkage and selection operator [LASSO], boosting, and multivariate adaptive regression splines [MuARS]) were each used to form a prognostic model for each of four outcomes obtained at a 12 month follow-up (disability—neck disability index [NDI]), quality of life (EQ5D), present neck pain intensity, and present arm pain intensity). For all four outcomes, the differences in mean performance between all four models were small (difference of NDI < 1 point; EQ5D < 0.1 point; neck and arm pain < 2 points). Given that the predictive accuracy of all four modelling methods were clinically similar, the optimal modelling method may be selected based on the parsimony of predictors. Some of the most parsimonious models were achieved using MuARS, a non-linear technique. Modern machine learning methods may be used to probe relationships along different regions of the predictor space.

## Introduction

Cervical radiculopathy (CR) is a prevalent disorder, and together with neck pain, ranks fourth in the burden of disease within the United States^1,2^. The natural history of CR is typically favourable^2^, and many patients can be initially treated conservatively^3^. However, those who fail to improve may be managed surgically^4^. Clinical prediction of outcomes in CR is paramount to facilitating optimal clinical decision making, managing patient expectations, and prioritising clinical efforts to individuals most at risk of poor recovery^5^.

Prognostic models play an important role not only in the clinical prediction of future health outcomes, but also identifying the most influential predictors that could inform either clinical management or lead to the development of novel therapeutic interventions^6^. Compared to other musculoskeletal disorders such as low back pain (LBP) and idiopathic neck pain (NP)^7–9^, there is comparatively fewer prognostic studies in the area of CR^10,11^. Current prognostic studies in CR have focused largely either on self-reported predictors^11,12^, or on objective physical measures^13^. Developing a prognostic model with both self-reported and physical measures could easily result in a model where the number of predictors exceed sample size and in this case, the model cannot be estimated with traditional fitting methods (e.g. maximum likelihood for simple regression) without additional penalisation as the corresponding algorithm for parameter estimation suffers from identifiability issues.

A typical statistical modelling strategy used when there are a large numbers of predictors is to first reduce the predictor subspace by conducting multiple univariate analysis, than enter the remaining predictors into a stepwise regression procedure^14^. There have been strong arguments against the traditional use of p-values in stepwise regression as a predictor selection technique. First, the regression coefficients are biased high in absolute value after model selection^15^. Second, the resulting p-values are based on invalid distribution assumptions and may yield overoptimistic prediction results^15^. Biased regression coefficients will result in the ensuing model having variable predictive performances when applied to different datasets.

Ideally, statistical methods that simultaneously perform predictor selection and penalized model fitting should be used when developing prognostic models with high numbers of predictors – such as the least absolute shrinkage and selection operator (LASSO)^16^, boosting^17^, and multivariate adaptive regression splines (MuARS)^18^. Increasingly, researchers are turning towards such machine learning techniques with built-in predictor selection functionality for developing accurate and parsimonious models (i.e. as few predictors as possible to achieve the best predictive accuracy)^19–21^. However, machine learning techniques for prognostic modelling has not been routinely used thus far in musculoskeletal pain research including CR. Whether more advance machine learning techniques offer superior performance over traditional statistical methods in the prediction of outcomes in individuals with CR is therefore unknown.

The primary aim of the present study is to compare the overall accuracy and the variability of prediction performance when developing parsimonious prognostic models of long-term recovery in individuals with CR across four domains of health: neck pain intensity, arm pain intensity, disability, and quality of life. The primary hypothesis was that traditional stepwise regression would result in the least accurate and greatest variability in predictive performance compared to techniques which perform automatic predictor selection (LASSO, boosting, and MuARS). The secondary aim of the study was to identify the most important predictors (i.e. predictors retained after variable selection) of recovery in individuals with CR post-surgery across the four mentioned health domains.

## Results

Descriptive characteristics of participants with complete data, included participants with missing data, and excluded participants with missing data are included in Table 1.

**Table 1:** Participant and pain characteristics of study cohort..

Variables	Complete (n = 71)	Missing_exclude (n = 8)	Missing_include (n = 122)	Total (n = 201)	P value
Group					0.787
Standard	33 (46.5%)	4 (50.0%)	63 (51.6%)	100 (49.8%)	
Structured	38 (53.5%)	4 (50.0%)	59 (48.4%)	101 (50.2%)	
Sex					0.570
Male	37 (52.1%)	5 (71.4%)	61 (50.8%)	103 (52.0%)	
Female	34 (47.9%)	2 (28.6%)	59 (49.2%)	95 (48.0%)	
Age (years)					0.035
Mean (SD)	51.986 (8.379)	48.750 (8.908)	48.779 (8.261)	49.910 (8.426)	
NDI_12m					0.401
Mean (SD)	11.296 (8.561)	6.571 (5.062)	11.095 (9.427)	10.972 (8.839)	
Vas_neck_now_12m					0.304
Mean (SD)	22.775 (24.282)	9.286 (9.268)	19.078 (24.542)	20.444 (23.984)	
Vas_arm_now_12m					0.348
Mean (SD)	22.254 (28.203)	7.625 (15.352)	20.525 (26.442)	20.664 (26.931)	
Vas_neck_now baseline					0.259
Mean (SD)	57.873 (22.601)	68.125 (25.284)	54.650 (25.275)	56.367 (24.384)	
Vas_arm_now baseline					0.407
Mean (SD)	50.662 (25.879)	63.375 (34.727)	49.456 (29.344)	50.477 (28.328)	
NDI baseline					0.469
Mean (SD)	19.887 (6.898)	23.375 (10.141)	20.730 (8.541)	20.537 (8.055)	

*Complete* individuals with complete data, *Missing_exclude* individuals with missing data and excluded from analysis, *Missing_include* individuals with missing data and included in analysis, *NDI* neck disability index, *Vas_neck(arm)_now_12m* current neck (arm) pain intensity at 12mth follow up, *Vas_neck(arm)_now baseline* current neck (arm) pain intensity at baseline.

**Figure 1:**
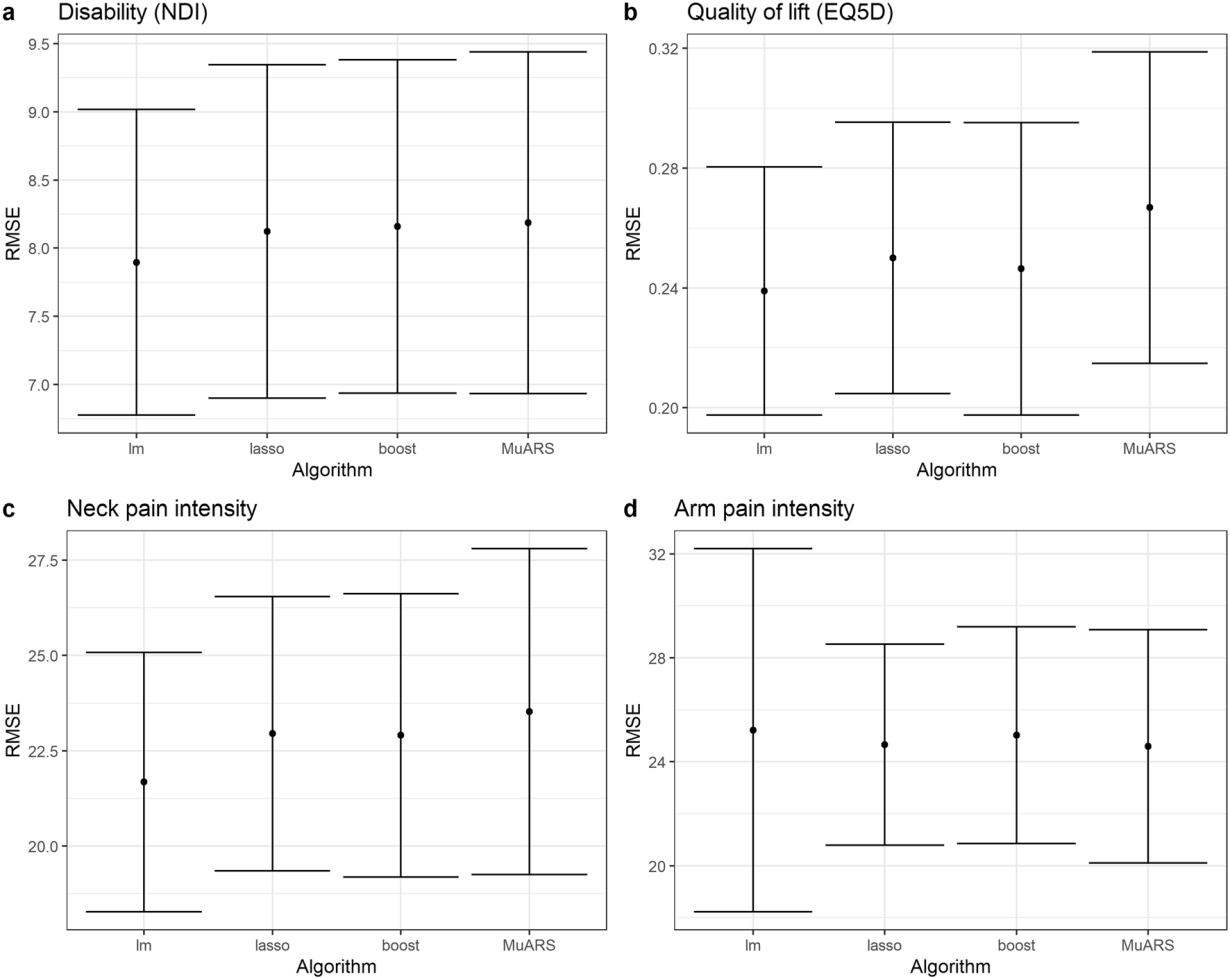
Accuracy and variability of predictive performance. *RMSE* root mean squared error, *NDI* neck disability index, *MuARS* multivariate adaptive regression spline, *lm* linear regression, *LASSO* least absolute shrinkage and selection operator.

The difference in mean accuracy of performance between models for the outcomes of neck disability index (NDI) (F = 5.371, *p* = 0.001), EuroQol five dimensions self-report (EQ5D) (F = 35.488, *p* < 0.001), and neck pain intensity (F = 22.36, *p* < 0.001) were significant (Fig. 1). For the outcome NDI, stepwise regression was the most accurate technique compared to least absolute shrinkage and selection operator regression (LASSO) (*p* = 0.028), boosting (*p* = 0.008), and multivariate adaptive regression splines (MuARS) (*p* = 0.002). For EQ5D, stepwise regression was the most accurate compared to LASSO (*p* = 0.001) and boosting (*p* = 0.042); whilst MuARS was the least accurate compared to stepwise regression (*p* < 0.001), LASSO (*p* < 0.001), and boosting (*p* < 0.001). For neck pain intensity, stepwise regression was the most accurate technique compared to LASSO (*p* < 0.001), boosting (*p* < 0.001), and MuARS (*p* < 0.001); whilst MuARS was also significantly less accurate than boosting (*p* = 0.04). For all four outcomes, the differences in mean performance between all four models were small (difference of NDI < 1 point; EQ5D < 0.1 point; neck and arm pain intensity < 2 points).

The difference in variability of performance between models for the outcomes of EQ5D (F = 5.651, *p* = 0.001), neck pain intensity (F = 8.575, *p* < 0.001), and arm pain intensity (F = 33.961, *p* < 0.001) were significant (Fig. 1). For the outcome EQ5D, stepwise regression was the least variable technique compared to boosting (*p* = 0.036) and MuARS (*p* = 0.001). For neck pain intensity, MuARS was the most variable technique compared to stepwise regression (*p* < 0.001), LASSO (*p* = 0.001) and boosting (*p* = 0.006). For arm pain intensity, stepwise regression was the most variable technique compared to LASSO (*p* < 0.001), boosting (*p* < 0.001) and MuARS (*p* < 0.001).

The coefficients of the best model for each outcome are presented in Table 2; with the remaining models included in the supplementary material (Supplementary material 1). For NDI at 12 months, baseline NDI was a common predictor selected across all four models. Given that the MuARS model is a non-linear method, the predictive influence of baseline NDI on NDI at 12 months only occurred if the baseline values were > 9. If baseline values of NDI were ≤ 9 the regression coefficients were zero. For EQ-5D at 12 months, baseline somatic perception (MSPQ) was a common predictor across all four models. For the MuARS model, the predictive influence of baseline MSPQ on EQ5D at 12^th^ months only occurred if the baseline value was > 26.

**Table 2:** Coefficients (in original units) of the selected predictors of the most accurate models for each outcome.

Outcome—NDI		Outcome—EQ5D		Outcome—Neck pain		Outcome—Arm pain	
Stepwise reg		Stepwise reg		Stepwise reg		LASSO	
Predictor	Coef	Predictor	Coef	Predictor	Coef	Predictor	Coef
(Intercept)	19.650	(Intercept)	0.590	(Intercept)	24.710	(Intercept)	30.700
NDI	0.484	MSPQ	− 0.008	NDI	0.892	Vas_arm_worst	0.103
C6_touch_r.1	− 3.260	SES	0.002	C7_pin_r.1	− 14.340	NDI	0.245
C8_pin_r.1	− 3.450	AROM_F	− 0.004	Reflex_triceps_r.1	7.750	MSPQ	0.062
Reflex_ach_r.1	− 4.340	Sx.2	− 0.120			EQ5D	− 3.583
		C7_pin_r.1	0.100			AROM_E	0.060
		Strn_fingabd_r.1	0.110			AROM_RR	− 0.108
						HRA_R	0.086
						Handst_r	− 0.095
						Romberg	0.040
						Figure 8	0.100
						CSQ_COP	1.155
						C5_touch_r.1	− 0.860
						C6_touch_r.1	− 13.040
						C7_pin_r.1	− 0.200

*Reg* regression, *LASSO* least absolute shrinkage and selection operator, *Coef* coefficient, *NDI* neck disability index, *C6(C5)_touch_r.1* C6(C5) level light touch on right normal, *C8(C7)_pin_r.1* C8(7) level pinprick on right normal, *Reflex_ach (triceps)_r.1* Achilles (triceps brachii) muscle reflex on right normal, *MSPQ* modified somatic perception questionnaire, *SES* self efficacy scale, *AROM_F(E/RR)* cervical flexion(extension/right rotation) active range of motion, *Sx.2* posterior cervical foraminotomy (PCF) with or without laminectomy, *Strn_fingabd_r.1* strength of finger abductors on right normal, *Vas_arm_worst* worst arm pain intensity, *EQ5D* quality of life, *HRA_R* head reposition accuracy from right to neutral, *Handst_r* right hand grip strength, *CSQ_COP* coping strategies questionnaire, coping subscale.

For neck pain intensity at 12th months, baseline NDI was selected across all models. For the MuARS model, the predictive influence of baseline cervical right axial rotation range of motion (AROM_RR), cervical neck extension range of motion (AROM_E), and NDI occurred if the respective baseline values were < 54, > 36, and > 14, respectively. The predictors of AROM_R and AROM_E interacted with the Romberg test, whilst NDI interacted with right triceps reflex. For arm pain intensity at 12 months, the right C6 light touch test was a common predictor across all four models.

## Discussion

This study aimed to develop prognostic models of recovery in individuals with CR across the outcomes of disability, quality of life, neck pain intensity, and arm pain intensity measured 12 months post-surgery. Our primary hypothesis was partially supported—stepwise regression was the least variable predictive technique presently investigated for the outcome of quality of life at 12^th^ months, but the same technique was also the most variable technique for the outcome of arm pain intensity. Importantly, differences in predictive performance across all techniques are likely to be clinically insignificant, based on published clinically meaningful differences^22^. The secondary finding of the present study was that baseline NDI was a common predictor for the outcomes of NDI and neck pain intensity at 12 months whereas somatic awareness was a predictor of quality of life and the right C6 light touch test was a predictor of arm pain intensity.

The novelty of the presently applied methods warrants a discussion of the rationale behind the study’s approach. A predictive model can be developed using theory-driven (i.e. classical hypothesis testing) or data-driven (e.g. machine learning) methods^23^. Theory-driven methods fit a model based on a theory (assumption) of a probability distribution of an outcome that is dependent on a controlled set of fixed predictors^23,24^. In data-driven methods, the predictors are not fixed but are tuned by the outcome to maximize the predictive accuracy of the model^23^; the predictors are bound to (potentially complex) probability distributions. The present study did not perform statistical inference with the regression coefficients, given that most classical inference techniques do not account for the probabilistic nature of both the predictors and outcome, inherent in machine learning methods^24^. Even statistical inference after stepwise regression methods, has been acknowledged to be an invalid procedure^25^, justifying the exclusion of its use presently. In defence, the primary aim of the present study was to develop the most accurate predictive model (i.e. prognostic model research^6^), rather than infer the population probability distribution of the outcome given a predictor.

The present study used multiple statistical methods to develop multiple prognostic models, rather than a single method which is commonly used in most prognostic studies^11–13,26^. An issue with defining a single model is the assumption that it is true or at least optimal in some sense^27^. It is common practice when using machine learning to use multiple methods^21,28^, and either use the single best model, or combine multiple models into a “meta” model. The latter approach reduces the bias and variability in the performance a single model might have, and combines different effects of the predictors found by different methods^29,30^. If a statistical model represents a snapshot of an “expert” system, the importance of a predictor would be greater if selected by multiple models than a single model.

The performance of our models was comparable to previous machine learning prediction models developed in LBP (root mean squared error (RMSE)_pain_ = 20–25 /100 points; RMSE_%disability_ = 17–20%)^31^. Given that the predictive accuracy of all four modelling methods were clinically similar, the optimal modelling method may be selected based on the parsimony of predictors. Some of the most parsimonious models were achieved using MuARS, a non-linear technique (see Supplementary material 2). The simplest example of a non-linear predictor is the addition of a quadratic term (e.g. y = x + ${x}^{2}$), with the interpretation being that the relationship between a predictor and outcome differs with different values of the former. For the ${MuARS}_{NDI12m}$ model, the hinge function “h(ndi -9)” indicated that the predictive relationship of β = 0.587 was present only when baseline NDI > 9. Given that a 5–14 points on the NDI scale reflects mild disability^32^, the predictive value of NDI only appeared in individuals with greater than mild baseline disability. The non-linear relationship between baseline and outcomes may not be surprising given that previous studies reported different non-linear rates of recovery in disability with different baseline NDI scores in individuals with whiplash associated disorders (WAD)^33^. To the authors knowledge, existing prognostic modelling studies in the musculoskeletal literature have only considered linear relationships, which may not accurately reflect for the potential non-linearity of physiological pain processes^34^.

The present study found that several local and global neuromuscular indices were predictive of disability, such as balance (Romberg), neck flexor and extensor endurance, “figure of 8” timing, cervical ROM, and cervical proprioceptive acuity. The present findings were supported by the literature which reported up to one-third of post-operative individuals with CR present with deficits in neck muscle strength and endurance at a 12^th^ month follow up, compared to healthy controls^35^. Previous studies have only reported the following baseline variables to be predictors of 12 month disability: pain intensity and psychological distress^12^; disability, axial cervical ROM, pain intensity, sex, hand grip strength^13^; axial cervical ROM and disability^11^. Considering the outcome of 24 months disability, the following predictors were selected in previous studies: worker’s compensation case and neurological sensory function^36^; pain intensity and cervical sagittal ROM^37^; disability, pain intensity, cervical sagittal and axial ROM, sex and hand-grip strength^13^; sex, and number of operated levels on the cervical spine^26^.

Paradoxically, better balance interacted with better cervical ROM, to predict worse neck pain at 12^th^ months using MuARS. This was in contrast to prior research which reported greater recovery in individuals with CR with better cervical ROM^11,37^. Based on the predictor of “h(54-AROM_RR) * h(Romberg- 12)”, a 1 s increase in Romberg timing and a 1° increase in right cervical rotation, increased neck pain by 0.127 points, only in individuals with poorer balance (< 12 s) but with better right cervical rotation (> 54°). Based on the predictor of “h(AROM_E-36) * h(12-Romberg)”, a 1 s increase in Romberg timing and a 1° increase in cervical extension, increased neck pain by 0.096 points, only in individuals with better balance (> 12 s) but with poorer cervical extension mobility (< 36°).

It is clinically more intuitive that better physical function is related to better prognosis, given that enhanced function is the aim of many therapeutic efforts. In the wider musculoskeletal literature, there have been reports of paradoxical relationships between physical function and pain, such as: (1) greater spine mobility predicting poorer recovery in back pain^38^, (2) greater physical activity levels increasing the risk of spinal pain onset^39^, and (3) greater hip internal rotation mobility, as one factor, increasing the responsiveness to manual therapy in back pain^40^. The predictors selected in the present study should not be interpreted causally but be restricted to the predictive framework. A causal understanding of any biopsychosocial variables with pain, quality of life and disability would require another type of statistical approach, such as mediation analysis^41^.

The present study has several strengths. Firstly, we included a holistic set of predictors that included physical, psychological, neurological, demographic variables. Second, we followed best practice guidelines in the development, validation, and report of our models^6,42^. Of note, we used resampling methods to achieve a more conservative estimate of our model performance. Third, the complexity of the presented models is alleviated through the provision of the codes and results of the present study, which means that readers can fully replicate the findings presently reported. A limitation of the present study is the small sample size relative to the number of predictors included, which precluded splitting our data into training and validation sets, the latter for independent validation^6^. In defence, the present study represents one of the largest prospective clinical investigations of individuals with CR, compared to previous research^10,11^. A previous simulation study reported that machine learning methods are “data hungry” – in that they may need 10 times as many events per predictor to achieve stable prediction within the classification framework^43^. Training models using low bias (i.e. highly accurate) techniques on small datasets, such as random forests, runs the risk of having highly variable predictive performance when generalizing to external contexts. Therefore, using methods with a higher bias (i.e. less accurate) is a strategy to build more conservative models on small datasets, to reduce potential performance variability. Strong regularization based on a penalized estimation as done in the LASSO and boosting, or rigorous variable selection, as done by stepwise selection procedures, may help further mitigate performance variability. In the present study we have therefore chosen methods that we think allowed for enough flexibility while considering the relatively small sample size.

## Conclusion

Baseline NDI was a common predictor for the outcomes of NDI and neck pain intensity; somatic awareness for the outcome of quality of life; and right C6 light touch test for the outcome of arm pain intensity. Although the present study did not observe clinically meaningful alterations in predictive accuracy and variability between models, given the relatively small ratio of sample size to predictors of the present study, it should not be automatically concluded that there is no role of modern machine learning methods in developing prognostic models. Interestingly, some of the most parsimonious models created have inherent non-linear characteristics, which supports the use of multiple machine learning methods to probe relationships along different regions of the predictor space. Future prognostic research would benefit from the findings of the present study on the more important predictors of recovery in CR, and use our methods on large sample sized cohorts to build prognostic models which balances accuracy, variability in performance, and model parsimony.

## Methods

### Study design

This is a prospective cohort study where the data were collected from a randomized controlled trial, methodological details of which have been previously reported^44–46^. All participants provided written informed consent, and the regional ethics review board in Linköping (Dnr M126-08) approved this study. All methods are reported in accordance with the Transparent Reporting of a multivariable prediction model for Individual Prognosis or Diagnosis (TRIPOD) guideline^42^.

### Participants

Participants with CR were recruited from four spinal surgery centres in the south of Sweden, if they fulfilled the inclusion criteria: aged 18–70 years old, persistent CR symptoms ≥ 2 months, magnetic resonance imaging results of disc disease commensurate with clinical findings, and unsatisfactory improvement after rehabilitation. The exclusion criteria were: cervical fracture or traumatic subluxation, previous neck surgery, cervical myelopathy, spinal malignancy, spinal infection, any disorders which precluded safe performance of an extensive rehabilitation program, myofascial pain syndromes, persistent severe LBP, diagnosis of a severe psychiatric disorder, drug or alcohol addiction, and power command of the Swedish language^46^.

### Interventions

A total of 201 participants (mean [standard deviation (SD)] age = 50.0 [8.4] years, males = 105, females = 96) were recruited. Participants were randomly allocated to either a structured or a standard (control) rehabilitation group prior to the operation^44–46^. The type of surgery received by each participant was individually determined by the surgeons at each of the four spinal centres, based on the patient’s clinical presentation^45^; 38 participants received a posterior cervical foraminotomy (PCF) with or without laminectomy, whilst 163 participants received an anterior cervical discectomy and fusion (ACDF).

#### Common post-surgical care (weeks 1 to 6 post-surgery)

All participants followed an identical rehabilitation pathway for the first six weeks immediately post-surgery^44,45^. Management included advice about appropriate ergonomics and posture, instructions about shoulder mobility exercises, and movements to avoid during the first post-surgical week. Patients returned for a routine visit 6 weeks post operation to the spinal centre with the surgeon; and a physiotherapist who instructed patients on neck mobility exercises. In some cases the contact with the surgeon at 6 weeks was conducted by a telephone call.

#### Structured post-surgical rehabilitation (weeks 7 to 26)

Participants were referred to a local primary care physiotherapist. Each physiotherapist received a half day training session with the project leader on the rehabilitation program. The structured program included a cervical neuromuscular and endurance training component and a cognitive-behavioural component^46^. Participants visited the physiotherapists once per week between weeks 7 to 12, and twice per week between weeks 13 to 26. Participants were also advised on the performance of a home exercise program. After week 26, participants were discharged and encouraged to continue increasing their physical activity levels.

#### Standard post-surgical rehabilitation (weeks 7–26)

Participants in this group were treated in accordance with the Swedish standard post-surgical care of individuals with CR. Briefly, participants were referred to their local physiotherapist on an as-needed basis, decided by the patients themselves. Any interventions were pragmatic and not designed to rehabilitate known neuromuscular deficits of neck pain disorders.

### Outcome variables

Four outcomes were used to define recovery—perceived disability (NDI^32^), perceived quality of life (EQ5D^47^), present neck pain intensity, and present arm pain intensity, all obtained at a 12 month follow-up. Details of the outcome measures can be found in the supplementary material (Supplementary material 2).

### Potential predictors

Predictors that were considered, included baseline collected demographic details (e.g. age), treatment group (structured vs standard rehabilitation), neurological sensory tests (e.g. light touch), neurological motor tests (manual muscle testing), neurological reflex tests, special musculoskeletal tests (e.g. Spurling’s), neurodynamic tests (upper limb neural tension), whole-body functional tests (e.g. Romberg balance), cervical neuromuscular assessment (e.g. neck muscle endurance), self-reported pain intensities, disability, quality of life, and psychological assessments (e.g. self-efficacy). Details of all the predictors, their original and transformed scales can be found in the supplementary material (Supplementary material 2).

### Data pre-processing and missing data handling

**Figure 2:**
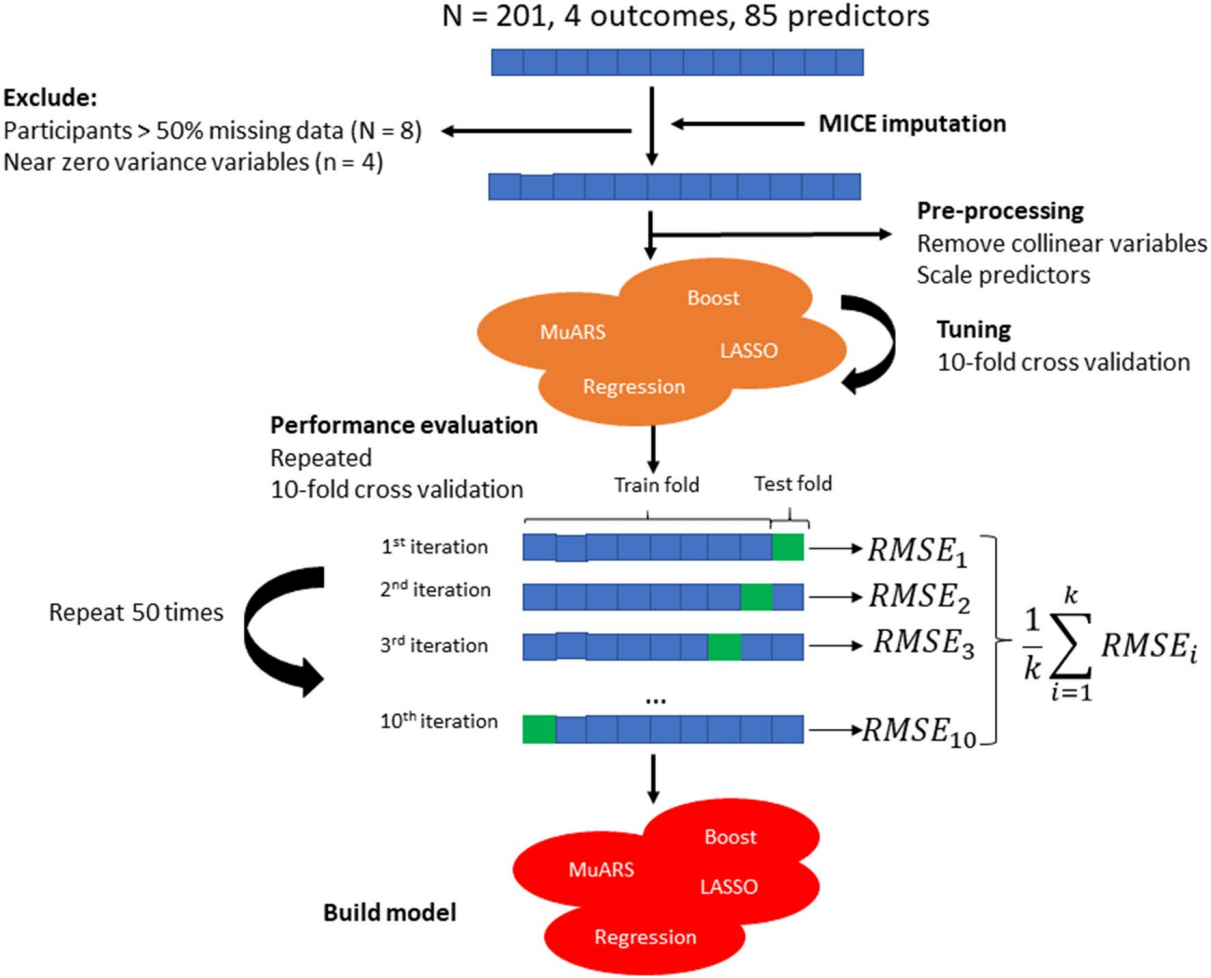
Predictive modelling workflow. *RMSE* root mean squared error, *MuARS* multivariate adaptive regression spline, *lm* linear regression, *LASSO* least absolute shrinkage and selection operator, *MICE* multivariate imputation by chained equations.

The workflow for analyses is illustrated in Fig. 2. Four variables, bilateral straight leg raise neurodynamic tests, and bilateral Babinski tests, were excluded as they demonstrated near zero variance (i.e. values remained near constant) across all participants^48^. Levels within categorical variables with relatively few participants were collapsed, such that the transformed levels each had a relatively balanced number of participants (see Supplementary material 2).

We performed several analyses to determine the appropriateness of missing value imputation. First, we performed Little’s missing completely at random (MCAR) test, to determine if values were missing (completely) at random. Second, we compared the main baseline characteristics of participants with and without missing data, to determine if there were any clinically relevant differences between groups. Participants with more than 50% missing data were also excluded (n = 8 participants).

Multiple imputation was performed on all predictor and outcome variables with missing values using the Multivariate Imputation by Chained Equations method^49^. We used the Random Forest algorithm for imputation as it was capable of imputing continuous and categorical variables, with a maximum iteration number of 1000.

### Prognostic modelling

The codes used for the present study are included in the supplementary material (results in compressed file also in Supplementary material 3). A total of 193 participants, 81 predictors, and four outcomes were used for modelling. Four modelling techniques were used for each outcome, yielding a total of 16 models. The following common modelling steps were followed for all approaches (Fig. 1). First, highly collinear continuous predictors were removed using a threshold > 0.7^50^. Second, all continuous predictors were scaled (demeaned and divided by its standard deviation [SD]) so that variables of different scales could have equal opportunity to be included into the model. For each of the four modelling techniques, the following tuning procedures were performed:

#### Two stage stepwise linear regression

First, potential predictors were singularly entered into simple linear regression models^14^. Predictors with a statistically significant relationship with the outcome, set at an alpha of 10%, were retained. Second, the retained variables were used in a multiple variable linear regression model. A bidirectional stepwise selection process was applied, where predictors with a p-value > 0.05 was removed, until only significant (*p* < 0.05) predictors remained^14^. Significant predictors remaining in this stage are subsequently used to build and validate the final linear regression model.

#### LASSO regression

LASSO regression constitutes a penalized linear model with a shrinkage penalty that induces sparsity of predictors in the model^42,51^. Due to the L1-penalty used by the LASSO, the effects of predictors can be shrunk to be zero, effectively resulting in predictor selection and thereby also improving prediction performance. For a given amount of shrinkage, as determined by the $\lambda$ value, the model can be estimated using coordinate descent (see “Algorithms” in Supplementary material 1). The optimal amount of shrinkage induced by the algorithm is found via a tenfold cross-validation (CV)^51^, and this $\lambda$ value is subsequently used to build and validate the final LASSO model.

#### Model-based boosting

Model-based boosting uses a component-wise gradient boosting algorithm for model fitting (see “Algorithms” in Supplementary material 1)^52^. The algorithm adds a predictor iteratively to the model to “correct” the error made by the prior model. To estimate the optimal number of iterations, a tenfold CV was performed. Given its iterative nature, some predictors are never selected, meaning that this method automatically performs predictor selection. The optimal iteration number is subsequently used to build and validate the final boosting model.

#### MuARS

Multivariate adaptive regression splines are semi-parametric extensions of linear models to capture non-linear or interaction effects of predictors. It includes non-linearity and interactions by evaluating each covariate using basis functions. Three types of basis functions for each covariate are used: constant functions, hinge (“h”) functions (piece-wise linear functions on two intervals connected with one knot) and products of two or more hinge functions. The model is then built in an iterative manner considering those basis functions for each predictor in a forward-pass and then reducing the model in a backward step to avoid overfitting (see “Algorithms” in Supplementary material 1). The selected predictors and associated basis functions were subsequently used to build and validate the final MuARS model.

### Performance validation

For all methods, the optimal hyperparameters (for LASSO and boosting) or the optimal set of remaining predictors, were used to build the respective models at the validation stage. Given that the outcomes are continuous, an appropriate metric of model performance would be the RMSE, between the predicted and observed outcome. For all methods, validation was performed using tenfold CV repeated 50 times^53^. A tenfold CV iteratively splits the training set into 10 approximately equal folds, trains the model on 9 folds and evaluates the model’s performance (i.e. yielding a RMSE) on the 10th fold. Hence, performing 50 repeated tenfold CV would yield 50 sets of 10 RMSE values. A repeated tenfold CV reduces validation optimism, since a model would perform well on the data it was exactly trained on^42^.

### Statistical inference

The dependent variables were the mean and standard deviation (SD) of RMSE values across a single tenfold validation. Given that 50 repeats were performed, each model produced 50 sets of mean and SD values. The independent variable was the four modelling techniques (stepwise regression, LASSO, boosting, MuARS). Simple regression was performed on the independent and dependent variables, with pairwise post-hoc inference investigated where appropriate. Significance was determined at a threshold of *p* < 0.05.

## Supplementary information

Supplementary Information 1.

Supplementary Information 2.

Supplementary Information 3.
