# Supplementary material for: Clinical predictive modelling of post-surgical recovery in individuals with cervical radiculopathy: a machine learning approach
Source: Sci Rep. 2020 Oct 8;10:16782. doi: 10.1038/s41598-020-73740-7 (PMC7545179; doi:10.1038/s41598-020-73740-7)
Supplement: Supplementary file 1 — Supplementary Information 1. [file 41598_2020_73740_MOESM1_ESM.docx]

# Supplementary material

Contents

[Supplementary material 1](#_Toc38016672)

[Algorithms 2](#_Toc38016673)

[Algorithm for LASSO regression 2](#_Toc38016674)

[Algorithm for model-based boosting 3](#_Toc38016675)

[Algorithm for multivariate adaptive regression splines 4](#_Toc38016676)

[Model coefficients 5](#_Toc38016677)

[12^th^ month neck disability index outcome 5](#_Toc38016678)

[12^th^ month EQ5D outcome 6](#_Toc38016679)

[12th month neck pain outcome 7](#_Toc38016680)

[12^th^ month arm pain outcome 8](#_Toc38016681)

## Algorithms

### Algorithm for LASSO regression

| 1. Start with an initial guess for each of the *p* covariate effects. Set *i = 1.* 2. Fix all covariate effects but the *i*th. 3. Find the optimal solution for the *i*th covariate effect by solving the penalized least squares objective using soft-thresholding 4. Iterate Steps 2 and 3 for each covariate *i* = *2, …, p* |
| --- |

### Algorithm for model-based boosting

| 1. Starting with a null model which contains no covariates, but only an intercept. 2. Specify a set of base learners for each covariates. Base learners are small models, such as a linear model or regression spline, for each covariate. The simplest example of a base learner is a linear relationship between a covariate and a response. Denote the number of base learners by P and set m = 0. 3. Increase m by 1, where m is the number of iterations.    1. Compute the negative gradient of the loss function and evaluate it at previous iteration m-1. This yields the negative gradient vector, which becomes the response variable for 3(b).    2. Fit each P base learners to the negative gradient vector using penalized least squares estimation. The resulting P regression fits yield P vectors h^m,p^, p=1,…,P of predicted values, where each vector is an estimate of the negative gradient vector.    3. Select the base learner h^m,*^ that best minimizes the residual sum of squares (RSS) criterion.    4. Update f^m^ = f^m-1^ + *v* h^m,*^, where 0 < *v* ≤ 1 is a real-valued learning rate. 4. Iterate Steps 3 and 4 until m = m_stop_. |
| --- |

### Algorithm for multivariate adaptive regression splines

| 1. Starting with a null model which contains no covariates, but only an intercept. Specify the maximum number of model terms k as well as the maximum degree of interaction between covariates. Specify the threshold for loss improvement e.  2. Forward step: Increase the model complexity by  a) Find the model term that improves the model most with respect to the highest residual sum-of-squares (RSS) reduction by considering  i) terms already in the model for interaction with other terms  ii) covariates not in the model to be included as a new term  and find the optimal knot for the newly added hinge function within all data points of the selected covariate.  b) Repeat step a) until the model consists of k terms or the RSS does not improve more than e in compared to the previous step.  3. Backward step: Reduce the model complexity  a) by comparing the current best model with all submodels that are obtained when removing one model term. The model with the best generalized cross-validation score (GCV) is defined as the new best model.  b) Repeat a) until no better model can be found. |
| --- |

## Model coefficients

### 12^th^ month neck disability index outcome

| LASSO | |  | Boosting | |  | MuARS | |
| --- | --- | --- | --- | --- | --- | --- | --- |
| Predictor | **Coef** |  | **Predictor** | **Coef** |  | **Predictor** | **Coef** |
| (Intercept) | 14.520 |  | Intercept | 11.000 |  | (Intercept) | 4.070 |
| Age | -0.008 |  | Age | -0.020 |  | h(NDI- 9) | 0.587 |
| NDI | 0.292 |  | NDI | 0.278 |  |  |  |
| MSPQ | 0.148 |  | MSPQ | 0.138 |  |  |  |
| SES | -0.035 |  | SES | -0.037 |  |  |  |
| EQ5D | -0.377 |  | AROM_RR | -0.022 |  |  |  |
| AROM_F | 0.018 |  | HRA_R | -0.027 |  |  |  |
| AROM_E | 0.018 |  | HRA_L | 0.021 |  |  |  |
| AROM_RR | -0.051 |  | Romberg | 0.072 |  |  |  |
| HRA_R | -0.080 |  | NME_F | -0.002 |  |  |  |
| HRA_L | 0.076 |  | CSQ_CAT | -0.025 |  |  |  |
| Romberg | 0.096 |  | Sx.2 | 1.837 |  |  |  |
| NME_F | -0.003 |  | C7_pin_r.1 | -0.932 |  |  |  |
| CSQ_CAT | -0.053 |  | C8_pin_r.1 | -0.577 |  |  |  |
| Sx.2 | 0.490 |  | Strn_del_l.1 | 0.347 |  |  |  |
| C6_touch_r.1 | -0.950 |  | Reflex_triceps_r.1 | 0.265 |  |  |  |
| C7_pin_r.1 | -1.190 |  |  |  |  |  |  |
| C8_pin_r.1 | -1.500 |  |  |  |  |  |  |
| Reflex_triceps_r.1 | 0.170 |  |  |  |  |  |  |
| Reflex_ach_r.1 | -1.280 |  |  |  |  |  |  |
| Abbreviations: Reg – regression; LASSO – least absolute shrinkage and selection operator; Coef – coefficient; NDI – neck disability index; C6(C5)_touch_r.1 - C6 level light touch on right normal; C8(C7)_pin_r.1 – C8(7) level pinprick on right normal; Reflex_ach (triceps)_r.1 – Achilles (triceps brachii) muscle reflex on right normal; MSPQ – modified somatic perception questionnaire; SES – self efficacy scale; AROM_F(E/RR) – cervical flexion(extension/right rotation) active range of motion; Sx.2 - posterior cervical foraminotomy (PCF) with or without laminectomy; NME_F – cervical flexor muscle endurance; EQ5D – quality of life; HRA_R(L) – head reposition accuracy from right (left) to neutral; CSQ_CAT – coping strategies questionnaire, catastrophizing subscale; Strn_deltoid_r.1 – strength of deltoids on right normal. | | | | | | | |

### 12^th^ month EQ5D outcome

| LASSO | |  | Boosting | |  | MuARS | |
| --- | --- | --- | --- | --- | --- | --- | --- |
| Predictor | **Coef** |  | **Predictor** | **Coef** |  | **Predictor** | **Coef** |
| (Intercept) | 0.640 |  | Intercept | 0.728 |  | (Intercept) | 0.82 |
| Vas_neck_best | 0.000 |  | NDI | -0.002 |  | h(MSPQ- 26) | -0.01 |
| NDI | -0.003 |  | MSPQ | -0.004 |  |  |  |
| MSPQ | -0.005 |  | SES | 0.001 |  |  |  |
| SES | 0.001 |  | EQ5D | 0.094 |  |  |  |
| EQ5D | 0.094 |  | AROM_F | -0.001 |  |  |  |
| AROM_F | -0.002 |  | AROM_RL | -0.002 |  |  |  |
| AROM_E | -0.001 |  | HRA_L | -0.002 |  |  |  |
| AROM_RR | 0.001 |  | Handst_r | -0.001 |  |  |  |
| AROM_RL | -0.002 |  | Romberg | -0.002 |  |  |  |
| HRA_L | -0.006 |  | CSQ_COP | -0.001 |  |  |  |
| Handst_r | -0.002 |  | Sx.2 | -0.079 |  |  |  |
| Romberg | -0.002 |  | C4_touch_l.1 | -0.004 |  |  |  |
| CSQ_COP | -0.007 |  | C7_pin_r.1 | 0.036 |  |  |  |
| Sx.2 | -0.060 |  | C7_pin_l.1 | -0.004 |  |  |  |
| C4_touch_l.1 | -0.010 |  | Reflex_triceps_r.1 | 0.265 |  |  |  |
| C6_touch_r.1 | 0.010 |  |  |  |  |  |  |
| C7_pin_r.1 | 0.060 |  |  |  |  |  |  |
| C7_pin_l.1 | -0.010 |  |  |  |  |  |  |
| C8_touch_r.1 | 0.010 |  |  |  |  |  |  |
| Strn_fingabd_r.1 | 0.050 |  |  |  |  |  |  |
| Reflex_ach_r.1 | 0.020 |  |  |  |  |  |  |
|  |  |  |  |  |  |  |  |
| Abbreviations: Reg – regression; LASSO – least absolute shrinkage and selection operator; Coef – coefficient; NDI – neck disability index; C6(C4/C8)_touch_r.1 - C6(C4/C8) level light touch on right normal; C7_pin_r(l).1 – C7 level pinprick on right (left) normal; Reflex_ach (triceps)_r.1 – Achilles (triceps brachii) muscle reflex on right normal; MSPQ – modified somatic perception questionnaire; SES – self efficacy scale; AROM_F(E/RR/RL) – cervical flexion(extension/right rotation/right lateral flexion) active range of motion; Sx.2 - posterior cervical foraminotomy (PCF) with or without laminectomy; HRA_L – head reposition accuracy from left to neutral; Handst_r – right hand grip strength; CSQ_COP – coping strategies questionnaire, coping subscale; Strn_fingabd_r.1 – strength of finger abductors on right normal; Vas_neck_best – lowest neck pain intensity; EQ5D – quality of life | | | | | | | |

### 12th month neck pain outcome

| LASSO | |  | Boosting | |  | MuARS | |
| --- | --- | --- | --- | --- | --- | --- | --- |
| Predictor | **Coef** |  | **Predictor** | **Coef** |  | **Predictor** | **Coef** |
| (Intercept) | 25.050 |  | Intercept | 20.031 |  | (Intercept) | 6.670 |
| Age | -0.006 |  | NDI | 0.436 |  | h(54-AROM_RR) * h(Romberg- 12) | 0.127 |
| NDI | 0.478 |  | EQ5D | -0.058 |  | h(AROM_E- 36) * h(12-Romberg) | 0.096 |
| MSPQ | 0.124 |  |  |  |  | h(NDI- 14) * Reflex_triceps_r.1 | 1.211 |
| EQ5D | -4.966 |  |  |  |  |  |  |
| AROM_E | 0.020 |  |  |  |  |  |  |
| AROM_RR | -0.029 |  |  |  |  |  |  |
| HRA_R | 0.274 |  |  |  |  |  |  |
| HANDST_R | -0.049 |  |  |  |  |  |  |
| Romberg | 0.022 |  |  |  |  |  |  |
| NME_F | -0.009 |  |  |  |  |  |  |
| NME_E | -0.003 |  |  |  |  |  |  |
| C6_touch_r.1 | -2.770 |  |  |  |  |  |  |
| C7_pin_r.1 | -4.180 |  |  |  |  |  |  |
|  |  |  |  |  |  |  |  |
| Abbreviations: Reg – regression; LASSO – least absolute shrinkage and selection operator; Coef – coefficient; NDI – neck disability index; C6_touch_r.1 - C6 level light touch on right normal; C7_pin_r.1 – C7 level pinprick on right normal; Reflex_triceps_r.1 – triceps brachii muscle reflex on right normal; MSPQ – modified somatic perception questionnaire; AROM_E(RR) – cervical extension (right rotation) active range of motion; HRA_R – head reposition accuracy from right to neutral; Handst_r – right hand grip strength; NME_F(E) – cervical flexor (extensor) muscle endurance; EQ5D – quality of life | | | | | | | |

### 12^th^ month arm pain outcome

| Stepwise reg | |  | Boosting | |  | MuARS | |
| --- | --- | --- | --- | --- | --- | --- | --- |
| Predictor | **Coef** |  | **Predictor** | **Coef** |  | **Predictor** | **Coef** |
| (Intercept) | 37.060 |  | Intercept | 20.171 |  | (Intercept) | 37.73 |
| Vas_arm_worst | 0.179 |  | Vas_arm_worst | 0.111 |  | C6_touch_r.1 | -23.54 |
| Figure8 | 0.148 |  | NDI | 0.234 |  |  |  |
| C6_touch_r.1 | -22.630 |  | EQ5D | -1.499 |  |  |  |
|  |  |  | AROM_RR | -0.052 |  |  |  |
|  |  |  | Handst_r | -0.089 |  |  |  |
|  |  |  | Figure8 | 0.069 |  |  |  |
|  |  |  | CSQ_COP | 0.388 |  |  |  |
|  |  |  | Sx.2 | 3.441 |  |  |  |
|  |  |  | C6_touch_r.1 | -6.210 |  |  |  |
|  |  |  | C6_touch_l.1 | 2.784 |  |  |  |
|  |  |  | Reflex_triceps_r.1 | 0.941 |  |  |  |
|  |  |  |  |  |  |  |  |
| Abbreviations: Reg – regression; LASSO – least absolute shrinkage and selection operator; Coef – coefficient; Vas_arm_worst – worst arm pain intensity; NDI – neck disability index; C6_touch_r(l).1 - C6 level light touch on right (left) normal; EQ5D – quality of life; Reflex_triceps_r.1 – triceps brachii muscle reflex on right normal; MSPQ – modified somatic perception questionnaire; AROM_RR – cervical right rotation active range of motion; Sx.2 - posterior cervical foraminotomy (PCF) with or without laminectomy; CSQ_COP – coping strategies questionnaire, coping subscale | | | | | | | |
