# Supplementary material for: Clinical predictive modelling of post-surgical recovery in individuals with cervical radiculopathy: a machine learning approach
Source: Sci Rep. 2020 Oct 8;10:16782. doi: 10.1038/s41598-020-73740-7 (PMC7545179; doi:10.1038/s41598-020-73740-7)
Supplement: Supplementary file 3 — Supplementary Information 3. [file 41598_2020_73740_MOESM3_ESM.zip › suppl/3_model_codes.pdf]

Data analysis for CxRad prospective analysis

Prognostic modelling using regression

Import libraries

```r
rm(list = ls())

# Helper
library(tidyverse)
library (arsenal)
library (janitor)
library (magrittr)
library (cowplot)

# Table generator
library (flextable)
library (officer)

# Imputation
library (mice)
library (VIM)
library (BaylorEdPsych)

# Modelling
library (mlr)
library (caret)
library (glmnet)
library (mboost)
library (earth)
library (olsrr)

# Exploration
library (corrr)

# Feature engineering
library (rsample)
library (recipes)
library (dataPreparation)

# Parallel processing
library (parallelMap)

# Custom functions
source ("regress_functions2.R")
```

Import imputed data

```
outcome_var <- c("ndi_12m", "eq5d_12m" , "vas_neck_now_12m" , "vas_arm_now_12m")

df <- read.csv("data_impute.csv")

# remove highly collinear predictors. Threshold > 0.7

hc <- df %>%
  dplyr::select (-contains("12m")) %>%
  select_if(is.numeric) %>%
  cor(use = "pairwise.complete.obs") %>%
  findCorrelation( cutoff=0.7, names = TRUE)


df2 <- df[, !names (df) %in% hc]
```

Tune parameters, internal validate, model fitting, external validate

- Loop for all outcomes, and save data.

```
use_scaled_outcome = FALSE

for (n in seq_along(outcome_var)) {

  # select outcome
  outcome <- outcome_var [n]

  # Unscaled predictors
  df_predict <- df2 %>%
    dplyr::select (-outcome_var)

  # Unscaled outcomes
  df_outcome <- df2 %>%
    dplyr::select (outcome)

  # Build scales
  predict_scales <- build_scales(dataSet = df_predict  , verbose = TRUE)
  outcome_scales <- build_scales(dataSet = df_outcome , verbose = TRUE)

  # Scaled outcomes
  df_outcome_preproc <- fastScale(dataSet = df_outcome,
                                  scales = outcome_scales, verbose = TRUE)

  # Select scaled or unscaled outcomes
  if (use_scaled_outcome == FALSE) {

    use_outcome <- df_outcome %>% pull ()

  } else {
```

```r
  use_outcome <- df_outcome_preproc  %>% pull ()

}


# Scaled dataframe

df_preproc <- fastScale(dataSet = df_predict,
                              scales = predict_scales, verbose = TRUE) %>%
  mutate (outcome  = use_outcome) %>% # change train_outcome_preproc if use pre-processed outcomes
  dplyr::select (outcome, everything ()) %>%
  as.data.frame()

###############################################################################
# Univariate predictor selection
keep_var <- uni_var_select(data = df_preproc,
                                univar_thresh = 0.1,
                                outcome = outcome)

df_preproc_sub <- df_preproc [, names (df_preproc) %in% c("outcome", keep_var)] %>% as.data.frame()

mod.lm <- lm (outcome ~.,  data =  df_preproc_sub)

# Stepwise regression
df.proc_lm <- step_regress(mod = mod.lm,
                            train_data = df_preproc_sub)
# Scaled dataframe for lm
df_preproc_lm <- df.proc_lm$train_dat_preproc.lm

# Unscaled dataframe
df_unscale <- bind_cols (df_outcome, df_predict)%>%
  rename (outcome = outcome) %>%
  as.data.frame()

df_unscale_lm <- df2 [, names (df2) %in% c(outcome, df.proc_lm$include_var)] %>%
  rename (outcome = outcome) %>%
  as.data.frame()

###############################################################################
# MARS tuning

mars_tune_param <- mars_tune (data = df_preproc)
degree <- mars_tune_param$degree
nprune <- mars_tune_param$nprune

# Lasso tuning

lambda_min <- lasso_tune(data = df_preproc)

# Boost tuning

mstop <- boost_tune (data = df_preproc, max_mstop = 10000, nu = 0.001)
```

```r
# Make mlr tasks, learners, and internal validation
options(mlr.debug.seed = 456)
n_rep  <-  50
int_valid_list <- make_mlr_task_learn(train_penal_dat = df_unscale, # unscale as mlr scales data
                                       train_lm_dat = df_unscale_lm, # unscale as mlr scales data
                                      lambda_min = lambda_min,
                                      mstop = mstop,
                                      degree = degree,
                                      nprune = nprune,
                                      repeats = n_rep)

# Make model and external validation

ext_valid_list <- make_mlr_model_pred (data = int_valid_list)

# Save results

save(outcome,
  predict_scales,
  outcome_scales,
  df_preproc,
  keep_var,
  df_preproc_lm,
  df_unscale,
  df_unscale_lm,
  mars_tune_param,
  lambda_min,
  degree,
  nprune,
  mstop,
  int_valid_list,
  ext_valid_list,
  file = paste0 ("../data/res_",outcome, ".RData"))

rm (
  outcome,
  predict_scales,
  outcome_scales,
  df_preproc,
  keep_var,
  df_preproc_lm,
  df_unscale,
  df_unscale_lm,
  df_outcome,
  df_outcome_preproc,
  df_predict,
  df_preproc_sub,
  df.proc_lm,
  mars_tune_param,
  lambda_min,
  degree,
  nprune,
  mstop,
```

```
    use_outcome,
    int_valid_list,
    ext_valid_list)

}
```
