# Supplementary material for: Clinical predictive modelling of post-surgical recovery in individuals with cervical radiculopathy: a machine learning approach
Source: Sci Rep. 2020 Oct 8;10:16782. doi: 10.1038/s41598-020-73740-7 (PMC7545179; doi:10.1038/s41598-020-73740-7)
Supplement: Supplementary file 3 — Supplementary Information 3. [file 41598_2020_73740_MOESM3_ESM.zip › suppl/3_results_codes.pdf]

Data analysis for CxRad prospective analysis

Report results

Bernard Liew

18 May, 2020

Import libraries

```
rm(list = ls())

# Helper
library(tidyverse)
library (arsenal)
library (janitor)
library (magrittr)
library (cowplot)

# Table generator
library (flextable)
library (officer)

# Imputation
library (mice)
library (VIM)
library (BaylorEdPsych)

# Modelling
library (mlr)
library (caret)
library (glmnet)
library (mboost)
library (earth)
library (olsrr)

# Exploration
library (corrr)

# Inference
library (multcomp)
library (car)

# Feature engineering
library (rsample)
library (recipes)
```

```r
library (dataPreparation)

# Parallel processing
library (parallelMap)

# Custom functions
source ("regress_functions2.R")

knitr::opts_chunk$set(eval = FALSE)
```

Get performance of all four models

```r
outcome_var <- c("ndi_12m", "eq5d_12m" , "vas_neck_now_12m" , "vas_arm_now_12m")
n_rep <- 50
performance <- list()

for (n in seq_along(outcome_var)) {

  load(file = paste0 ("../data/res_",outcome_var[n], ".RData"))

  performance[[n]] <- get_sample_performance(data = int_valid_list,
                                             round2 = 2,
                                             outcome = outcome_var[n],
                                             n_fold = 10,
                                             n_rep = n_rep)

  rm (outcome,
    predict_scales,
    outcome_scales,
    df_preproc,
    keep_var,
    df_preproc_lm,
    df_unscale,
    df_unscale_lm,
    mars_tune_param,
    lambda_min,
    degree,
    nprune,
    mstop,
    int_valid_list,
    ext_valid_list)


}

legend <- get_legend(
  # create some space to the left of the legend
  performance[[1]]$fig.perm.iter +
    guides(color = guide_legend(nrow = 1)) +
    theme(legend.position = "bottom")
)
```

```
# f <- plot_grid(performance[[1]]$fig.perm.iter + theme(legend.position="none"),
#                performance[[2]]$fig.perm.iter + theme(legend.position="none"),
#                performance[[3]]$fig.perm.iter + theme(legend.position="none"),
#                performance[[4]]$fig.perm.iter + theme(legend.position="none"),
#                legend,
#                ncol = 2,
#                rel_heights = c(1, 1, .1),
#                labels =  c("a", "b", "c", "d", ""))
#
# tiff ("../manuscript/fig2_iter.tiff", width = 10, height = 8, units = "in", res = 300)
# f
# dev.off()


f <- plot_grid(performance[[1]]$fig.perm.ave + theme(legend.position="none") +
                 labs (title = "Disability (NDI)", x = "Algorithm"),
               performance[[2]]$fig.perm.ave + theme(legend.position="none") +
                 labs (title = "Quality of lift (EQ5D)", x = "Algorithm"),
               performance[[3]]$fig.perm.ave + theme(legend.position="none") +
                 labs (title = "Neck pain intensity", x = "Algorithm"),
               performance[[4]]$fig.perm.ave + theme(legend.position="none") +
                 labs (title = "Arm pain intensity", x = "Algorithm"),
               ncol = 2,
               labels =  c("a", "b", "c", "d"))

f

# tiff ("../manuscript/fig2_ave.tiff", width = 10, height = 8, units = "in", res = 300)
# f
# dev.off()
```

Statistical inference

Mean accuracy

Main effects

```
m_aov_list <- list()
post_hoc <- list()
post_hoc_df <- list()

for (n in seq_along(outcome_var)) {

  df <- performance[[n]]$perm.df %>%
    mutate (rep = rep (rep (c (1:n_rep), times = 10), times = 4)) %>%
    group_by(algorithm, rep) %>%
    summarise(Mean = mean (rmse))

  m <- lm (Mean ~ algorithm, data = df)
  m_aov_list[[n]] <- car::Anova(m, type = 3)
```

```
  post_hoc[[n]] <- summary(glht(m, linfct = mcp(algorithm = "Tukey")))
  post_hoc_df[[n]] <- data.frame (coef = post_hoc[[n]]$test$coefficients,
                                  p_val = post_hoc[[n]]$test$pvalues)

  if (m_aov_list[[n]][2, 4] >= 0.05) {
    sig = "insignificant"

  } else {

    sig = "significant"
  }

  cat ("The difference in mean accuracy of performance between models for the outcome of ",
       outcome_var[n], "was ", sig,  "(F = ", round (m_aov_list[[n]][2, 3], 3),
       ",", "P =", round (m_aov_list[[n]][2, 4], 3), ")", "\n")

}
```

Post-hoc

```
sig_str <- list()

for (n in seq_along(outcome_var)) {

  if (m_aov_list[[n]][2,"Pr(>F)"] < 0.05) {


    cat (paste ("for the outcome of", outcome_var[n], "the following contrasts were significant:"), "\n

    for (m in seq_along(rownames (post_hoc_df[[n]]))) {

      if (post_hoc_df[[n]][m, "p_val"] < 0.05) {

        coef <- round (post_hoc_df[[n]][m, "coef"], 3)
        p <- round (post_hoc_df[[n]][m, "p_val"], 3)
        xtrast <- rownames(post_hoc_df[[n]][m,])
        cat(paste (xtrast, "(difference = ", coef, ", P = ", p, ")"), "\n")
      }
    }
  }
}
```

Variability

Main effect

```
m_aov_list <- list()
post_hoc <- list()
post_hoc_df <- list()
```

```r
for (n in seq_along(outcome_var)) {

  df <- performance[[n]]$perm.df %>%
    mutate (rep = rep (rep (c (1:n_rep), times = 10), times = 4)) %>%
    group_by(algorithm, rep) %>%
    summarise(Sd = sd (rmse))

  m <- lm (Sd ~ algorithm, data = df)
  m_aov_list[[n]] <- car::Anova(m, type = 3)
  post_hoc[[n]] <- summary(glht(m, linfct = mcp(algorithm = "Tukey")))
  post_hoc_df[[n]] <- data.frame (coef = post_hoc[[n]]$test$coefficients,
                                  p_val = post_hoc[[n]]$test$pvalues)

  if (m_aov_list[[n]][2, 4] >= 0.05) {
    sig = "insignificant"

  } else {

    sig = "significant"
  }

  cat ("The difference in variability of performance between models for the outcome of ",
       outcome_var[n], "was ", sig,  "(F = ", round (m_aov_list[[n]][2, 3], 3),
       ",", "P =", round (m_aov_list[[n]][2, 4], 3), ")", "\n")

}
```

Post-hoc

```r
sig_str <- list()

for (n in seq_along(outcome_var)) {

  if (m_aov_list[[n]][2,"Pr(>F)"] < 0.05) {


    cat (paste ("for the outcome of", outcome_var[n], "the following contrasts were significant:"), "\n

    for (m in seq_along(rownames (post_hoc_df[[n]]))) {

      if (post_hoc_df[[n]][m, "p_val"] < 0.05) {

        coef <- round (post_hoc_df[[n]][m, "coef"], 3)
        p <- round (post_hoc_df[[n]][m, "p_val"], 3)
        xtrast <- rownames(post_hoc_df[[n]][m,])
        cat(paste (xtrast, "(difference = ", coef, ", P = ", p, ")"), "\n")
      }
    }
  }
}
```

Export coefficients

Read in saved data

```
rm(list = ls())

# Custom functions
source ("regress_functions2.R")

outcome_var <- c("ndi_12m", "eq5d_12m" , "vas_neck_now_12m" , "vas_arm_now_12m")

n <- 1

load(file = paste0 ("../data/res_",outcome_var[n], ".RData"))

use_scaled_outcome <- FALSE
```

Get model coefficients and rescale

```
coef.list <- get_coef_all_models(int_data = int_valid_list,
                                 ext_data = ext_valid_list,
                                 round2 = 10)
coef.list[[4]]
# get rescale coefficients

rescale_pred <- predict_scales %>%
  map (bind_cols) %>%
  bind_rows(.id = "predictors")

# rescale all coefficients

for (i in seq_along(names (coef.list))) {

  temp.df <- coef.list[[i]]

  for (f in seq_along(temp.df$predictors)) {

    for (m in seq_along(rescale_pred$predictors)) {

      if (grepl (rescale_pred$predictors[m],temp.df$predictors[f])){

        temp.df$coef[f] <-
          (temp.df$coef[f] / rescale_pred$sd[m])

      }

    }

    temp.df$coef[f] <- round ( temp.df$coef[f], 3)
```

```
  }

  coef.list[[i]] <- temp.df

}

# rescale mars hinge value

temp <- coef.list[[4]] %>%
  separate(predictors, into = c("var1", "var2"), sep = "\\*") %>%
  mutate(hinge1 = str_extract_all(var1, "[-+]?\\d+\\.\\d*") %>% as.numeric(),
         hinge2 = str_extract_all(var2, "[-+]?\\d+\\.\\d*")%>% as.numeric())

for (f in seq_along(temp$var1)) {

  for (m in seq_along(rescale_pred$predictors)) {

    if (grepl (rescale_pred$predictors[m],temp$var1[f])){

       temp$hinge1[f] <-
        round (((temp$hinge1[f] *
                   rescale_pred$sd[m]) +
                  rescale_pred$mean[m]), 3)
    }

  }

}


for (f in seq_along(temp$var2)) {

  for (m in seq_along(rescale_pred$predictors)) {

    if (grepl (rescale_pred$predictors[m],temp$var2[f])){

       temp$hinge2[f] <-
        round (((temp$hinge2[f] *
                   rescale_pred$sd[m]) +
                  rescale_pred$mean[m]), 3)
    }

  }

}



coef.list[[4]] <- temp %>%
  mutate (hinge1 = as.character(hinge1),
          hinge2 = as.character (hinge2)) %>%
  mutate(var1 = str_replace(var1,
                                   "[-+]?\\d+\\.\\d*", hinge1),
```

```
        var2 = str_replace(var2,
                                       "[-+]?\\d+\\.\\d*", hinge2)) %>%
  unite ("predictors", var1, var2, sep = "*", na.rm = TRUE) %>%
  dplyr::select (predictors, coef)

coef.list[[4]]
```

Export tables to word documents

```
model_type <- c("linear regression", "LASSO", "boosting", "MultAdaptRegSpline")
mlr_mod <- c("lm", "lasso", "boost", "mars")
file_names <- c("ndi", "eq5d" , "neckpain" , "armpain")

for (m in seq_along(mlr_mod)) {

  ft <- flextable(coef.list[[m]]) %>%
  set_header_labels(predictors = "Predictors",
                    coef = "beta") %>%
  set_caption(paste0("Coefficients for ", model_type[m], " model")) %>%
  bold(i = c(nrow(coef.list[[m]]))) %>%
  autofit()

  assign(paste0("ft.", mlr_mod[m]), ft)

}

my_path <- paste0("../manuscript/table_",
                  file_names[n],
                  ".docx")

my_doc <- read_docx()  %>%
  body_add_flextable(ft.lm) %>%
  body_add_break()%>%
  body_add_flextable(ft.lasso) %>%
  body_add_break() %>%
  body_add_flextable(ft.boost) %>%
  body_add_break() %>%
  body_add_flextable(ft.mars)

print (my_doc, target = my_path)
```
