# Supplementary material for: Clinical predictive modelling of post-surgical recovery in individuals with cervical radiculopathy: a machine learning approach
Source: Sci Rep. 2020 Oct 8;10:16782. doi: 10.1038/s41598-020-73740-7 (PMC7545179; doi:10.1038/s41598-020-73740-7)
Supplement: Supplementary file 3 — Supplementary Information 3. [file 41598_2020_73740_MOESM3_ESM.zip › suppl/regress_functions2.pdf]

Helper functions for analysis

Bernard Liew

18 May, 2020

```
data_split_proc <- function (data,
                             prop,
                             grouping,
                             outcome_var,
                             outcome,
                             use_scaled_outcome = FALSE) {

  # Split data into train and test

  dat_split <- initial_split(data, prop = prop, strata = grouping)
  train_dat <- training (dat_split)
  test_dat <- testing(dat_split)

  # Scaled predictors
  train_predict_preproc <- train_dat %>%
    dplyr::select (-outcome_var)

  test_predict_preproc <- test_dat %>%
    dplyr::select (-outcome_var)

  # Unscaled outcomes
  train_outcome <- train_dat %>%
    dplyr::select (outcome)

  test_outcome <- test_dat %>%
    dplyr::select (outcome)

  predict_scales <- build_scales(dataSet = train_predict_preproc , verbose = TRUE)
  outcome_scales <- build_scales(dataSet = train_outcome , verbose = TRUE)

  # Scaled outcomes
  train_outcome_preproc <- fastScale(dataSet = train_outcome,
                                     scales = outcome_scales, verbose = TRUE)

  test_outcome_preproc <- fastScale(dataSet = test_outcome,
                                    scales = outcome_scales, verbose = TRUE)

  if (use_scaled_outcome == FALSE) {

    use_train_outcome <- train_outcome %>% pull ()
    use_test_outcome <- test_outcome %>% pull ()
  } else {
```

```
    use_train_outcome <- train_outcome_preproc %>% pull ()
    use_test_outcome <- test_outcome_preproc %>% pull ()

  }


  # Combined predictors with outcomes
  train_dat_preproc <- fastScale(dataSet = train_predict_preproc,
                                 scales = predict_scales, verbose = TRUE) %>%
    mutate (outcome  = use_train_outcome) %>% # change train_outcome_preproc if use pre-processed outco
    dplyr::select (outcome, everything ()) %>%
    as.data.frame()

  test_dat_preproc <- fastScale(dataSet = test_predict_preproc,
                                scales = predict_scales, verbose = TRUE) %>%
    mutate (outcome = use_test_outcome) %>% # change train_outcome_preproc if use pre-processed outcome
    dplyr::select (outcome, everything ()) %>%
    as.data.frame()

  df_proc_list <- list (

    train_dat_preproc = train_dat_preproc,
    test_dat_preproc = test_dat_preproc,
    predict_scales = predict_scales,
    outcome_scales = outcome_scales
  )

  return (df_proc_list)

}




uni_var_select <- function (data, univar_thresh, outcome){

  # Follow example based on article http://dx.doi.org/10.1136/bjsports-2016-096084
  p_extract <- function (x) {

    p <- x[1, "Pr(>F)"]
    return (p)
  }

  # Univariate analysis
  uni_names <-  data %>%
    dplyr::select (-outcome) %>%
    map(~lm(data$outcome ~ .x, data = data)) %>%
    map(anova) %>%
    map_dbl(p_extract) %>%
    "<" (univar_thresh) %>%
    which() %>%
    names()
```

```r
  return (uni_names)

}

step_regress <- function (mod, train_data){


  # step.lm <- SignifReg(mod,
  #                      direction = "both",
  #                      criterion = "p-value",
  #                      correction = "None")

  step.lm <- olsrr::ols_step_both_p(mod.lm,
                                    pent = 0.05,
                                    prem = 0.05,
                                    details = FALSE)


  final.terms <- step.lm$predictors

  # yobs <-  train_data$outcome
  # yhat <- predict (step.lm)
  # rmse <- sqrt(mean((yhat - yobs)^2))

  #If scaled outcomes used, rescale error
  #rmse <- rmse *outcome_scales[[outcome]]$sd + outcome_scales[[outcome]]$mean

  train_dat_preproc.lm <- train_data[, names (train_data) %in% c("outcome", final.terms)]
  #test_dat_preproc.lm <- test_data[, names (test_data) %in% c("outcome", final.terms)]

  lm_tune_list <- list (#remove_var = rm_vars,
    include_var = final.terms,
    #predict_err = rmse,
    train_dat_preproc.lm = train_dat_preproc.lm
    #test_dat_preproc.lm = test_dat_preproc.lm
    )

  return  (lm_tune_list)

}


## Do lasso to get lambda

lasso_tune <- function (data) {

  y <- as.vector (data$outcome)

  x <- model.matrix (~ ., data[,!grepl ("outcome", names (data))]) [,-1]


  tunegrid <- 10^seq(10,-2,length=100)
```

```r
  cv <- cv.glmnet(x,
                  y,
                  alpha = 1,
                  lambda = tunegrid,
                  nfolds = 10,
                  standardize = FALSE,
                  intercept = TRUE)
  #plot(cv)
  lambda.min <- cv$lambda.min

  #coef(cv, s = cv$lambda.min)
  #cv$lambda.min

  return (lambda.min)
}


## Do boosting to get mstop

boost_tune <- function (data, max_mstop = 10000, nu = 0.001){

  mod.boost <- glmboost(outcome ~ .,
                        data = data,
                        control = boost_control(mstop = max_mstop, nu = nu),
                        center = FALSE)

  cv10f <- cv(model.weights(mod.boost), type = "kfold")
  cvm <- cvrisk(mod.boost, folds = cv10f)
  #plot (cvm)
  m <- mstop (cvm)

  return (m)

}

# Do mars to get degree and nprune

mars_tune <- function (data){

  tunegrid <- expand.grid(
    degree = 1:2,
    nprune = seq(2, 100, length.out = 50) %>% floor())

  marstune <- train (x = data[, !(names(data) %in% "outcome")],
                     y = data[["outcome"]],
                     method="earth",
                     metric = "RMSE",
                     tuneGrid = tunegrid, # tuning grid
                     trControl = trainControl(method = "cv", number = 10))


  degree <- marstune$bestTune$degree
  nprune <- marstune$bestTune$nprune
```

```r
  mars_tune <- list (degree = degree,
                     nprune = nprune)

  return (mars_tune)

}

make_mlr_task_learn <- function (train_penal_dat,
                                 train_lm_dat,
                                 lambda_min,
                                 mstop,
                                 degree,
                                 nprune,
                                 repeats) {

  # Make ml train task

  trtask.penal <-  makeRegrTask(id = "rad.penal",
                                data = train_penal_dat,
                                target = "outcome" ) %>%
    normalizeFeatures(method = "standardize") %>%
    createDummyFeatures(method = "reference")

  trtask.lm <-  makeRegrTask(id = "rad.lm",
                             data = train_lm_dat,
                             target = "outcome") %>%
    normalizeFeatures(method = "standardize") %>%
    createDummyFeatures(method = "reference")


  # Make lm learner

  lrn.lm <- makeLearner("regr.lm")


  # # lasso learner
  lrn.lasso <- makeLearner("regr.glmnet",
                           alpha = 1,
                           lambda = lambda_min,
                           intercept = TRUE,
                           standardize = FALSE)

  # make glmboost learner
  lrn.boost <- makeLearner("regr.glmboost",
                           family = "Gaussian",
                           center = FALSE,
                           mstop = mstop,
                           nu = 0.001)

  # make mars learner
  lrn.mars <- makeLearner("regr.earth",
                          degree = degree,
                          nprune = nprune) # maximum iteration
```

```r
  # Choose the resampling strategy
  rdesc = makeResampleDesc("RepCV", folds = 10, reps = repeats)

  r.lm <- resample(lrn.lm, trtask.lm, rdesc, measures = list( mlr::rmse))
  r.boost <- resample(lrn.boost, trtask.penal, rdesc, measures = list(mlr::rmse))
  r.lasso <- resample(lrn.lasso, trtask.penal, rdesc, measures = list(mlr::rmse))
  r.mars <- resample(lrn.mars, trtask.penal, rdesc, measures = list(mlr::rmse))

  learner.list <- list (lrn.lm = lrn.lm,
                        lrn.boost = lrn.boost,
                        lrn.mars = lrn.mars,
                        lrn.lasso = lrn.lasso)

  resampling.list <- list (r.lm = r.lm,
                           r.boost = r.boost,
                           r.lasso = r.lasso,
                           r.mars = r.mars)

  task.list <- list (trtask.penal = trtask.penal,
                     trtask.lm = trtask.lm)

  internal_validate <- list (task = task.list,
                             learner = learner.list,
                             resampling_error = resampling.list)

  return (internal_validate)

}


make_mlr_model_pred <- function (data) {

  mod.lm <-  mlr::train(data$learner$lrn.lm, data$task$trtask.lm)
  mod.boost <-  mlr::train(data$learner$lrn.boost, data$task$trtask.penal)
  mod.lasso <-  mlr::train(data$learner$lrn.lasso , data$task$trtask.penal)
  mod.mars <-  mlr::train(data$learner$lrn.mars, data$task$trtask.penal)


  mod.list <- list (mod.lm = mod.lm,
                    mod.boost = mod.boost,
                    mod.lasso = mod.lasso,
                    mod.mars = mod.mars)

  external_validate <- list (mod = mod.list#,
                             #pred = prediction.list
                             )

  return (external_validate)
}

get_sample_performance <- function (data,  round2, outcome, n_fold, n_rep){

  ##### Regression ############
```

```
df.lm.performance <-
  data.frame(error = rep ("rmse", n_fold*n_rep),
             algorithm = rep ("lm", n_fold*n_rep)) %>%
  bind_cols(round (data$resampling_error$r.lm$measures.test, round2))

###### Lasso #########

df.lasso.performance <-
  data.frame(error = rep ("rmse", n_fold*n_rep),
             algorithm = rep ("lasso", n_fold*n_rep)) %>%
  bind_cols(round (data$resampling_error$r.lasso$measures.test, round2))


####### Boosting ##############


df.boost.performance <-
  data.frame(error = rep ("rmse", n_fold*n_rep),
             algorithm = rep ("boost", n_fold*n_rep)) %>%
  bind_cols(round (data$resampling_error$r.boost$measures.test, round2))


###### Mars ###########


df.mars.performance <-
  data.frame(error = rep ("rmse", n_fold*n_rep),
             algorithm = rep ("MuARS", n_fold*n_rep)) %>%
  bind_cols(round (data$resampling_error$r.mars$measures.test, round2))


perm.df <- bind_rows(df.lm.performance,
                     df.lasso.performance,
                     df.boost.performance,
                     df.mars.performance) %>%
  mutate (algorithm = factor (algorithm, levels = c("lm", "lasso", "boost", "MuARS"))) %>%
  as.data.frame()

f <- ggplot (data = perm.df) +
  geom_point(aes(x = iter, y = rmse, colour = algorithm)) +
  geom_line (aes(x = iter, y = rmse, colour = algorithm)) +
  scale_colour_manual(values = c("black", "red", "blue", "darkgreen")) +
  labs (x = "iteration",
        y = "RMSE",
        title = outcome) +
  theme_bw()

perm.df.summ <- bind_rows(df.lm.performance,
                     df.lasso.performance,
                     df.boost.performance,
                     df.mars.performance) %>%
  mutate (algorithm = factor (algorithm, levels = c("lm", "lasso", "boost", "MuARS")))%>%
  group_by(algorithm)%>%
```

```
    summarise(Mean = mean (rmse),
              Sd = sd (rmse)) %>%
    ungroup() %>%
    as.data.frame()

  f1 <- ggplot (data = perm.df.summ) +
    geom_point(aes(x = algorithm, y = Mean)) +
    geom_errorbar(aes(x = algorithm, ymin = Mean - Sd, ymax = Mean + Sd)) +
    labs (x = "algoritm",
          y = "RMSE",
          title = outcome) +
    theme_bw()


  perm.list <- list (perm.df = perm.df,
                     fig.perm.iter = f,
                     fig.perm.ave =f1)


  return (perm.list)

}


get_coef_all_models <- function (int_data, ext_data, round2) {

  ##### Regression #############

  df.lm.performance <-
    data.frame(predictors = c("rmse"),
               coef = round (int_data$resampling_error$r.lm$aggr, round2))

  coef.df.lm <- coef(getLearnerModel(ext_data$mod$mod.lm)) %>%
    as.data.frame() %>%
    rename(coef = ".") %>%
    mutate (predictors = row.names(.),
            coef = round (coef, 2)) %>%
    dplyr::select (predictors, coef) %>%
    bind_rows(df.lm.performance)

  ###### Lasso #########

  df.lasso.performance <-
    data.frame(predictors = c("rmse"),
               coef = round (int_data$resampling_error$r.lasso$aggr, round2))


  coef.df.lasso <-coef(getLearnerModel(ext_data$mod$mod.lasso)) %>%
    as.matrix() %>%
    as.data.frame() %>%
    rename (coef = s0) %>%
    mutate (predictors = row.names(.),
            coef = round (coef, 2)) %>%
```

```
  dplyr::select (predictors, coef) %>%
  filter (coef != 0) %>%
  bind_rows(df.lasso.performance)


####### Boosting ##############


df.boost.performance <- data.frame(predictors = c("rmse"),
                                   coef = round (int_data$resampling_error$r.boost$aggr, round2))



coef.boost <- coef(getLearnerModel(ext_data$mod$mod.boost))

intercept <- data.frame (predictors = "Intercept",
                         coef = attr(coef.boost, "offset") %>% round (round2))

coef.boost <-   coef.boost %>%
  as.data.frame() %>%
  rename(coef = ".") %>%
  mutate (predictors = row.names(.),
          coef = round (coef, round2)) %>%
  dplyr::select (predictors, coef)

coef.df.boost <-  intercept %>%
  bind_rows (coef.boost) %>%
  bind_rows(df.boost.performance)


###### Mars ###########


df.mars.performance <- data.frame(predictors = c("rmse"),
                                  coef = round (int_data$resampling_error$r.mars$aggr, 2))


coef.df.mars <-coef(getLearnerModel(ext_data$mod$mod.mars))%>%
  as.data.frame() %>%
  rename(coef = ".") %>%
  mutate (predictors = row.names(.),
          coef = round (coef, 2)) %>%
  dplyr::select (predictors, coef) %>%
  bind_rows(df.mars.performance)

coef.list <- list (reg = coef.df.lm,
                   lasso = coef.df.lasso,
                   boost = coef.df.boost,
                   mars = coef.df.mars)

return (coef.list)
```

```
}
```
